# Identification of the key genes in children with sepsis by WGCNA in multiple GEO datasets

**DOI:** 10.3389/fped.2025.1518908

**Published:** 2025-05-16

**Authors:** Yue-chuan Shen, Dao-jun Yu, Ze Yu, Xue Zhao

**Affiliations:** ^1^ Zhejiang Chinese Medical University, Hangzhou, Zhejiang, China; ^2^ Department of Emergency, Zhoushan Hospital, Wenzhou Medical University, Zhoushan, China; ^3^ Department of Laboratory Medicine, Hangzhou First People's Hospital, the Fourth School of Clinical Medicine, Zhejiang Chinese Medical University, Hangzhou, Zhejiang, China; ^4^ The Laboratory of Cytobiology and Molecular Biology, Zhoushan Hospital, Wenzhou Medical University, Zhoushan, China; ^5^ Department of Emergency, Affiliated Hangzhou First People's Hospital, School of Medicine, Westlake University, Hangzhou, Zhejiang, China

**Keywords:** pediatric sepsis, WGCNA, GEO datasets, KEGG analysis, molecular mechanisms

## Abstract

Pediatric sepsis is a serious condition causing organ failure owing to immune dysregulation, linked to high morbidity and mortality, highlighting the need for quick detection and treatment. This study aims to identify key genes involved in pediatric sepsis using three gene expression datasets from the Gene Expression Omnibus. We first identified differentially expressed genes (DEGs) with R, then conducted a gene set enrichment analysis, and integrated DEGs with important module genes from weighted gene coexpression network analysis. We also screened adult sepsis datasets to find genes specific to pediatric cases, ultimately validating XCL1 as a key gene. This study suggests that XCL1 is crucial in understanding pediatric sepsis etiology and its molecular mechanisms.

## 1 Introduction

Sepsis is a life-threatening condition affecting all ages, marked by abnormal immune responses and organ dysfunction, posing a significant public health challenge (1). Globally, pediatric sepsis occurs in 22 cases per 100,000 person-years, and neonatal sepsis at 2,202 cases per 100,000 live births, totaling around 1.2 million cases annually (2, 3). The pediatric sepsis mortality rate is 25%, mainly due to refractory shock or organ dysfunction, with many deaths occurring within the first 48–72 h (4). Timely detection, appropriate resuscitation, and meticulous care are essential for optimizing the prognosis of children with sepsis (5).

Historically, sepsis was believed to primarily result from sustained inflammatory response to infection. However, clinical research aimed at treating sepsis by targeting key inflammatory molecules, either selectively or non-selectively, has not achieved significant progress (6). Most research revealed that sepsis development involves not only a prolonged and intense inflammatory response but also immunosuppression (7). This process involves a complex molecular network formed by interactions among cytokines, chemokines, and neuroendocrine factors.

In recent years, advanced analytical methods that leverage biological networks have emerged to extract key information from a broad range of histological data and to uncover the interactions present within this information (8). The main types are gene regulation, protein interactions, and coexpression networks. Weighted gene coexpression network analysis (WGCNA) helps reveal connections between gene clusters with similar expression in transcriptomic data and disease phenotypes, aiding in identifying molecular markers or therapeutic targets in complex diseases (9).

This study established a WGCNA network utilizing data from the Gene Expression Omnibus (GEO), encompassing peripheral whole blood samples from children with sepsis and healthy controls. Through the application of coexpression networks and diverse bioinformatics methodologies, this research elucidated modules and hub genes correlated with the prognosis of pediatric sepsis, with the objective of identifying potential biomarkers closely associated with clinical outcomes.

## 2 Materials and methods

### 2.1 Data sources and gene expression profiles

We searched the GEO database for high-throughput functional genomics studies on pediatric sepsis, finding relevant microarray datasets from children with sepsis and healthy controls, including GSE26378, GSE26440, GSE13904, and GSE131761 (10–13). The limma package was used for statistical analysis, error detection, data cleaning, and organization, improving data management. The robust multi-array average (RMA) method normalized data, and limma identified DEGs with *p* < 0.05 and log2 fold-change ≥1.

GSE26378, GSE26440, and GSE13904 are pediatric sepsis datasets, while GSE131761 is for adults. An analysis of four gene expression datasets was conducted, with clinical details given in Supplementary Table S1. The analysis included GSE26378, GSE26440, and GSE13904, excluding GSE131761, to identify sepsis-related genes in children (10–13).

### 2.2 WGCNA analysis and module identification

The WGCNA method improves gene set expression analysis using the WGCNA R package for constructing gene networks. Cluster analysis identifies outliers, while an automated system creates coexpression networks. Modules undergo functional assessment via hierarchical clustering and dynamic tree cutting, with Module Membership (MM) and Gene Significance (GS) evaluated for clinical associations. Central modules have high MM correlation and a *p*-value of 0.05, with MM over 0.80 and GS above 0.1 indicating strong connectivity and significance. Gene information for these modules supports further research.

Genes within the clinically significant gene module network with a GS value exceeding 0.2 and an MM value greater than 0.80 are classified as hub genes. Genes identified as overlapping are chosen as candidates for pivotal roles. The Venn R package is employed to produce significant gene diagrams.

### 2.3 Supplementary method

Supplementary data provide the details on the methods for identifying DEGs, conducting functional enrichment analysis, and performing GeneMANIA analysis (14).

## 3 Results

### 3.1 Identification of DEGs in these datasets

**Figure 1:**
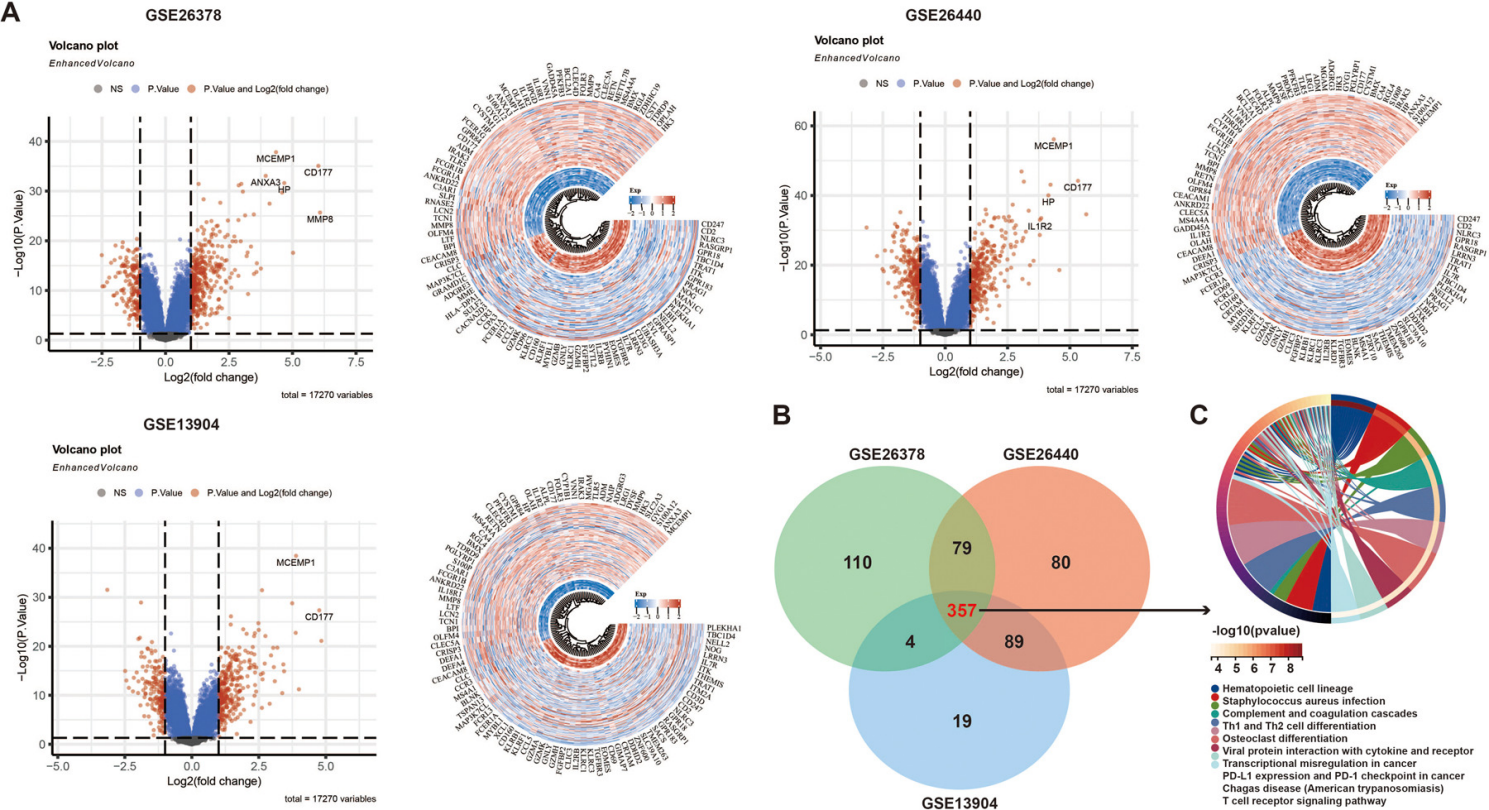
Identification of DEGs in three datasets: **(A)** volcano and heatmaps of differential genes in three GEO datasets, **(B)** a Venn diagram of the DEGs, and (**C**) KEGG analysis of the DEGs.

Figure 1A identified differentially expressed genes (DEGs) in the GSE26378, GSE26440, and GSE13904 datasets using *p* < 0.05 and |log2 (fold-change)| > 1, revealing their roles in immune responses, especially neutrophil activation and cytokine production (Supplementary Figures S1–S3). By analyzing the overlapping regions of DEGs through a Venn diagram, we identified 357 common gene regions (Figure 1B). Subsequently, GO and KEGG analyses were conducted on these overlapping genes. The findings demonstrated that these genes were primarily enriched in the biological processes associated with T-cell differentiation and PD-L1 expression (Figure 1C, Supplementary Figure S4).

### 3.2 Identification of coexpression gene modules in pediatrics sepsis

**Figure 2:**
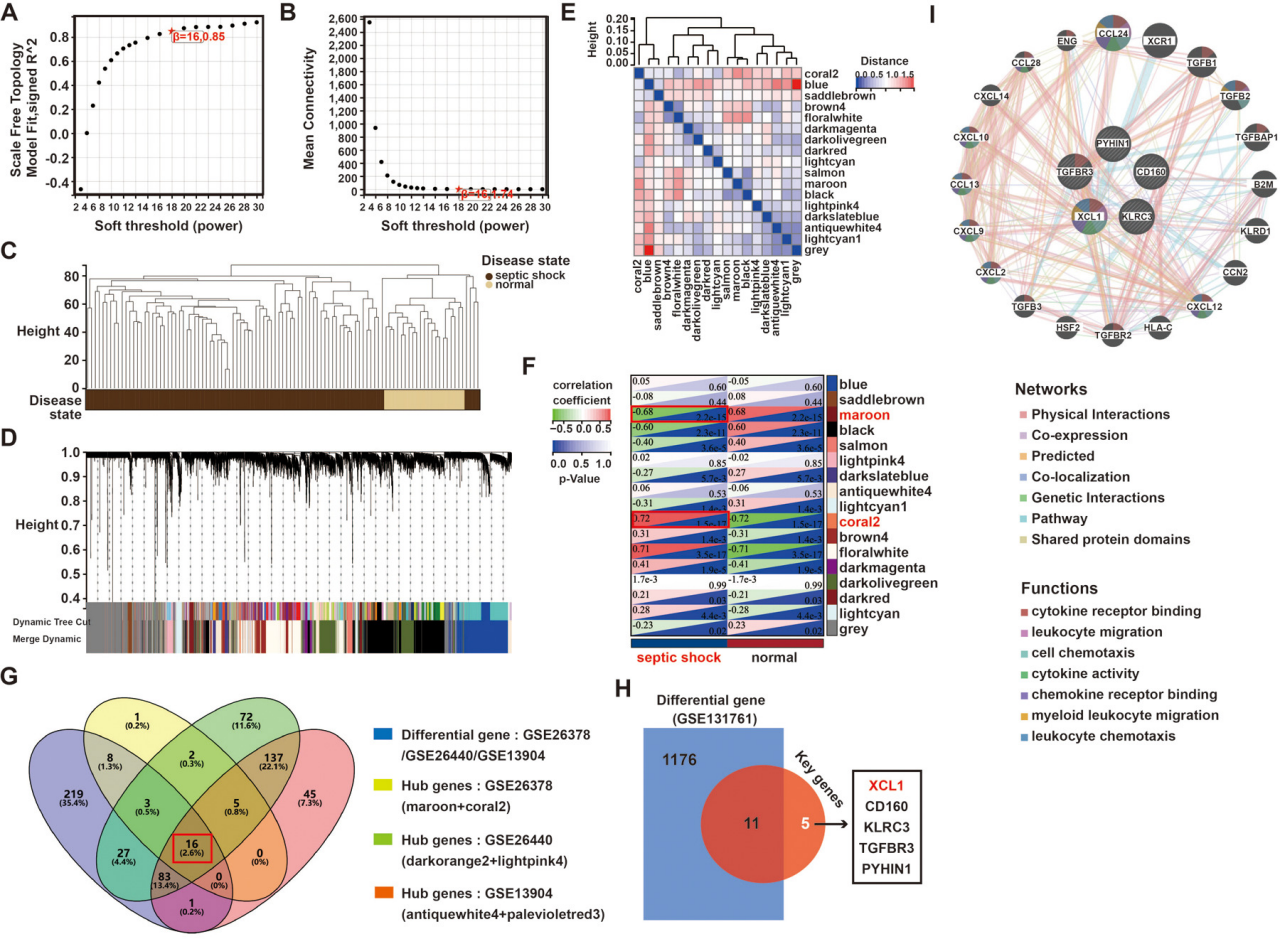
A WGCNA analysis of the pediatric sepsis and health conditions in the GSE26378 dataset: **(A,B)** an analysis of network topology for various soft thresholds (*β*), **(C)** a clustering dendrogram of all samples, **(D)** the gene dendrograms obtained by average linkage hierarchical clustering, **(E)** a heatmap depicts a TOM of genes selected for weighted coexpression network analysis, **(F)** module–trait relationships, **(G)** a Venn diagram of key module genes vs. DEGs, **(H)** a Venn diagram of the DEGs from GSE131761 and the 16 hub genes, and **(I)** GeneMANIA was used to analyze the function and correlation of hub 5 genes of pediatric sepsis.

We used weighted WGCNA to find coexpression gene modules in the pediatric sepsis dataset GSE26378, selecting a soft-threshold power of *β* = 16 for a scale-free network with a scale independence value of above 0.85 (Figures 2A,B). Samples from the GSE26378 dataset was classified into the pediatric sepsis group and the control group, with no outliers detected (Figure 2C). Hierarchical clustering and dynamic branch cutting techniques were employed on the gene dendrogram, leading to the identification of 17 modules (Figures 2D,E). The heatmap displays the topological overlap matrix (TOM) of the analyzed genes. The analysis demonstrated a high degree of independence among the modules associated with gene expression (Figure 2E). The brown module (indicative of positive correlation) and coral2 module (indicative of negative correlation) were significantly correlated with pediatric sepsis and selected for further examination (Figure 2F). In terms of module membership, these two modules encompass 35 genes significantly linked to pediatric sepsis.

At the same time, we used the same method to analyze the hub modules and genes of two other GSE datasets (GSE26440 and GSE13904). As shown in Supplementary Figures S5, S6, in the GSE26440 dataset, hub modules lightpink4 and darkorange2 contained a total of 345 genes, while in the GSE13904 dataset, hub modules antiquewhite4 and palevioletred3 contained a total of 287 genes. Further, based on the 357 DEGs obtained previously, the hub genes came from the three datasets, and a Venn analysis revealed 16 intersected genes, including CD160, XCL1, and CLIC3 (Figure 2G).

### 3.3 Identification of pediatric sepsis–related hub genes

Furthermore, to examine the specificity of these 16 genes in pediatric sepsis, we also used another adult sepsis dataset (GSE131761), whose patient clinical information was also presented in Supplementary Table S1. In the GSE131761 dataset, 559 genes were upregulated and 628 were downregulated in adult sepsis (Supplementary Figure S7).

After an intersection analysis of the 1,187 DEGs and 16 hub genes of pediatric sepsis, ultimately, 5 specific hub genes of pediatric sepsis were found, namely XCL1, CD160, KLRC3, TGFBR3, and PYHIN1 (Figure 2H). In addition, another adult sepsis dataset (GSE46955) was also used to analyze the hub genes. The difference analysis of the five core genes showed that only XCL1 and CD160 had no significant difference in adult sepsis and healthy people, so XCL1 and CD160 were candidates for the hub genes in pediatric sepsis (Supplementary Figure S8). Finally, a GeneMANIA analysis revealed that XCL1 had the most interaction among the five genes involved in a variety of cell signal transduction, including cytokine receptor binding, leukocyte migration, cytokine activity, and cellular chemotaxis (Figure 2I).

## 4 Discussion

Sepsis is a dysregulated response to infections and is common in children with various illnesses. Pediatric sepsis has a better prognosis than in adults, but risks remain (15). Children undergo rapid growth and immune changes from birth to adolescence, affecting their responses to respiratory infections (16). Treatment guidelines for sepsis underscore the necessity for the prompt administration of antibiotics in children exhibiting a high suspicion of sepsis, to enhance prognosis (17). Identifying diagnostic markers and immune cell patterns in pediatric sepsis is crucial for optimizing prognosis and understanding its immune impact. This endeavor will deepen our comprehension of the impact of pediatric sepsis on the immune system.

WGCNA identifies genes with similar expression profiles and organizes them into modules, suggesting interconnected functions and shared signaling pathways (18). WGCNA improves coexpression analysis by removing strict thresholds, preserving key biological information (19).

We used blood gene expression data linked to pediatric sepsis to create a coexpression network, identifying modules associated with sepsis and highlighting the gene XCL1, which was significantly elevated in affected children. This discovery not only offers new insights into the pathogenesis of pediatric sepsis but also establishes a foundation for future investigations into related diagnostic and therapeutic strategies.

XCL1 is produced by T, NK, and NKT cells during infections and inflammation, playing a key role in these processes and linked to diseases such as infections, autoimmune disorders, and tumors (20). Some research studies show XCL1 expression increases in various infections, notably in activated CD8+ T cells during chronic tuberculosis in mice, indicating a link to pathogenesis (21, 22). In a model of experimental pneumococcal meningitis, XCL1 and other cytokines have been detected during the acute phase of infection (23). Furthermore, XCL1 expression is similarly elevated in mice with chronic infections such as cytomegalovirus and herpes simplex virus (24, 25). In autoimmune diseases, XCL1 expression is also heightened. It can be identified in the synovial tissue of patients with rheumatoid arthritis, with elevated levels observed in tissue samples from sarcoidosis and Crohn's disease (26–28). The increased expression of XCL1 is crucial to the development and pathogenesis of inflammatory neurological diseases, including multiple sclerosis and HTLV-1-associated myelopathy (HAM) (25). These findings underscore the significant role of XCL1 in a range of infections and autoimmune diseases.

The methodology of the study involved an independent analysis of three data sets, highlighting the need to address batch effects when integrating multiple datasets to avoid biased results. Integration can be achieved using methods like ComBat or Harmony, with changes in batch effects visualized through principal component analysis (PCA) or t-distributed stochastic neighbor embedding (t-SNE). Conclusions are drawn from data integration without batch correction, indicating potential technical variations. Future research should validate key findings through independent cohorts or sensitivity analysis.

Our research findings suggested that XCL1 was the hub gene in pediatric sepsis. This study is not without limitations, as the conclusions remain invalidated through animal models or clinical samples. In addition, the conclusions of this study are based on a retrospective public data set analysis, which has not been validated in an independent cohort, especially in prospective children with sepsis, and may be limited by the heterogeneity of the original data (e.g., treatment regimens, ethnic differences) and technical bias (e.g., interplatform batch effects). We hope to obtain a multicenter pediatric SEPSIS cohort through international cooperation (such as the European Sepsis database and American PHIS database). Subsequent validation experiments should cover different age stages (neonates/children), pathogen types (bacteria/viruses), and sepsis phenotypes (shock/non-shock) to assess the broad applicability of markers.

In the future, we will undertake systematic investigations, including the assessment of XCL1 expression levels in children with sepsis and the correlation with clinical characteristics (e.g., disease severity), thereby exploring the function of XCL1 in pediatric sepsis. By employing *in vitro* cell models and small animal models, we will comprehensively explore the biological roles of XCL1 in pediatric sepsis, analyzing how XCL1 modulates the immune response and pathological progression of sepsis through the activation and chemotaxis of immune cells, including T cells, NK cells, and macrophages.

## 5 Conclusions

The pathogenesis of sepsis is intricate and not yet completely elucidated; however, it is marked by a sustained, excessive inflammatory response and a disturbance in intraorganismal homeostasis that is difficult to restore. To identify molecular targets reflective of pediatric sepsis pathology, it is crucial to reevaluate our research methodologies and promote interdisciplinary collaboration, particularly between medicine and fields such as computer science, to advance innovation in the diagnosis and prevention of pediatric sepsis.

## Data Availability

The original contributions presented in the study are included in the article/Supplementary Material, further inquiries can be directed to the corresponding authors.
