# Supplementary material for: Identification of the key genes in children with sepsis by WGCNA in multiple GEO datasets
Source: Front Pediatr. 2025 May 16;13:1518908. doi: 10.3389/fped.2025.1518908 (PMC12122430; doi:10.3389/fped.2025.1518908)
Supplement: Supplementary file 1 [file Datasheet1.docx]

**Supplementary Figures**

**Supplementary Figure 1**

**
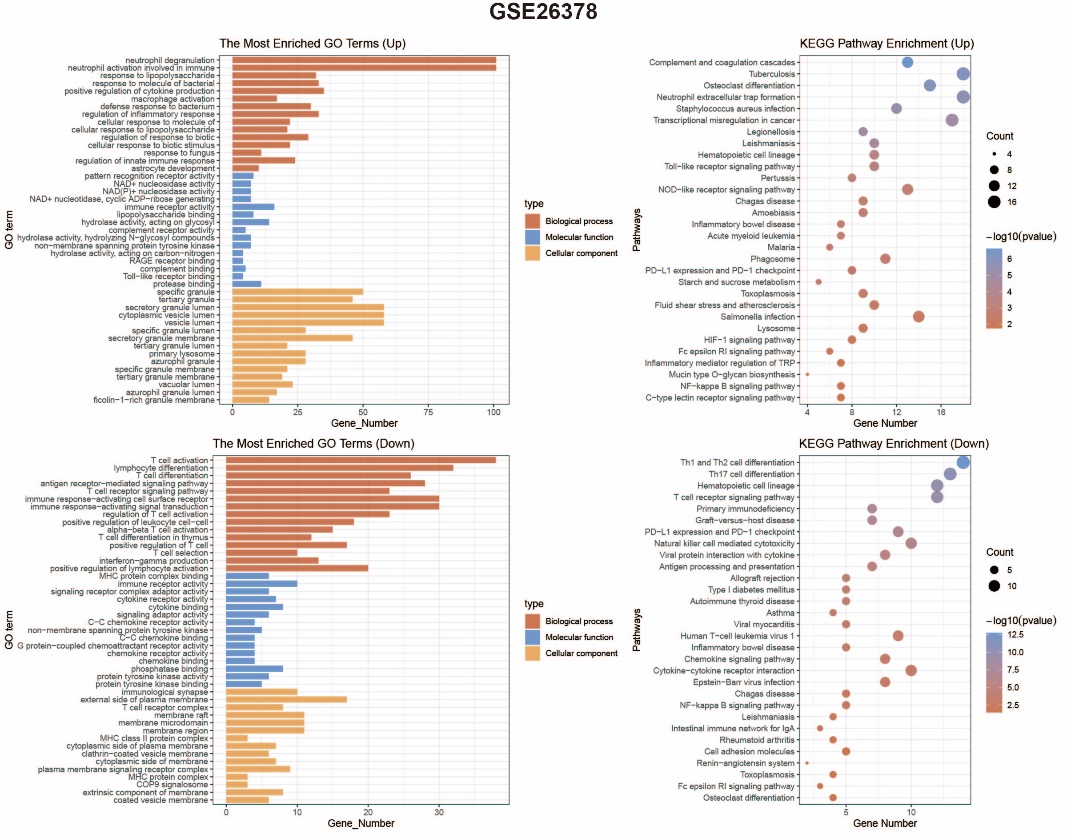
**

**Supplementary Figure 1** Functional enrichment analysis included KEGG pathway enrichment and GO term enrichment results of differential genes (**GSE26378**).

**Supplementary Figure 2**


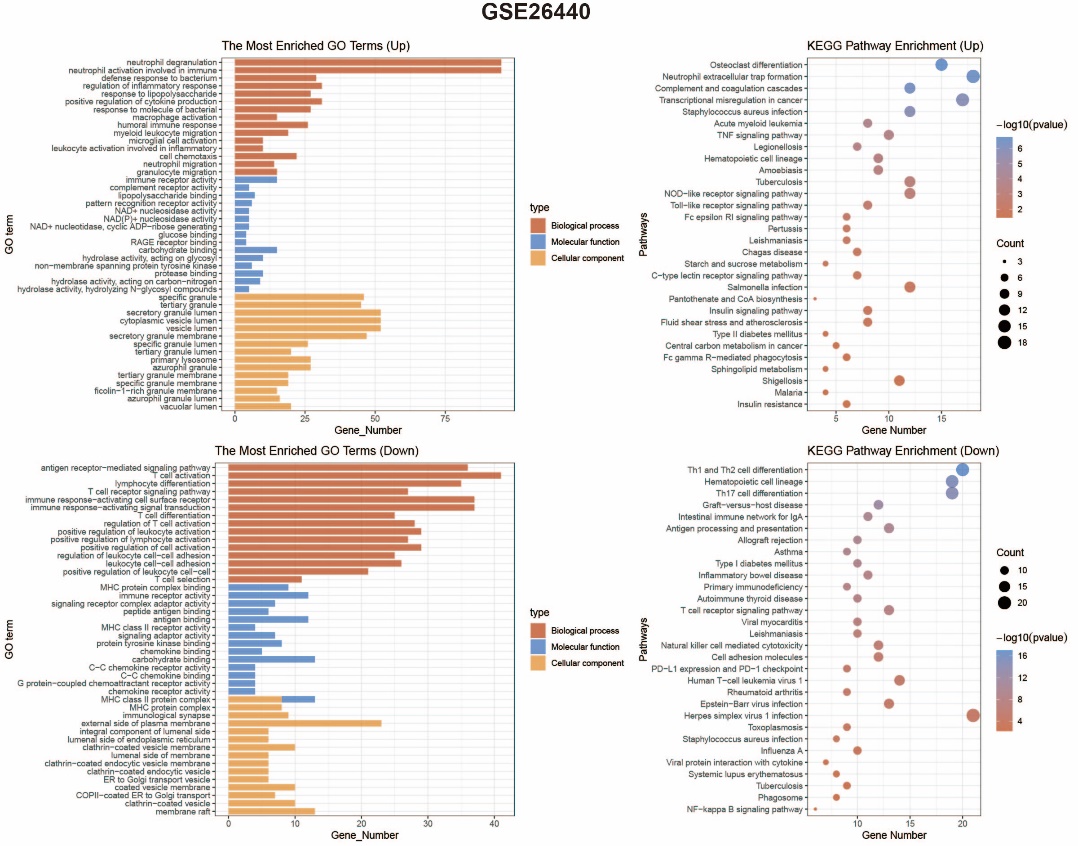


**Supplementary Figure 2** Functional enrichment analysis included KEGG pathway enrichment and GO term enrichment results of differential genes (**GSE26440**).

**Supplementary Figure 3**


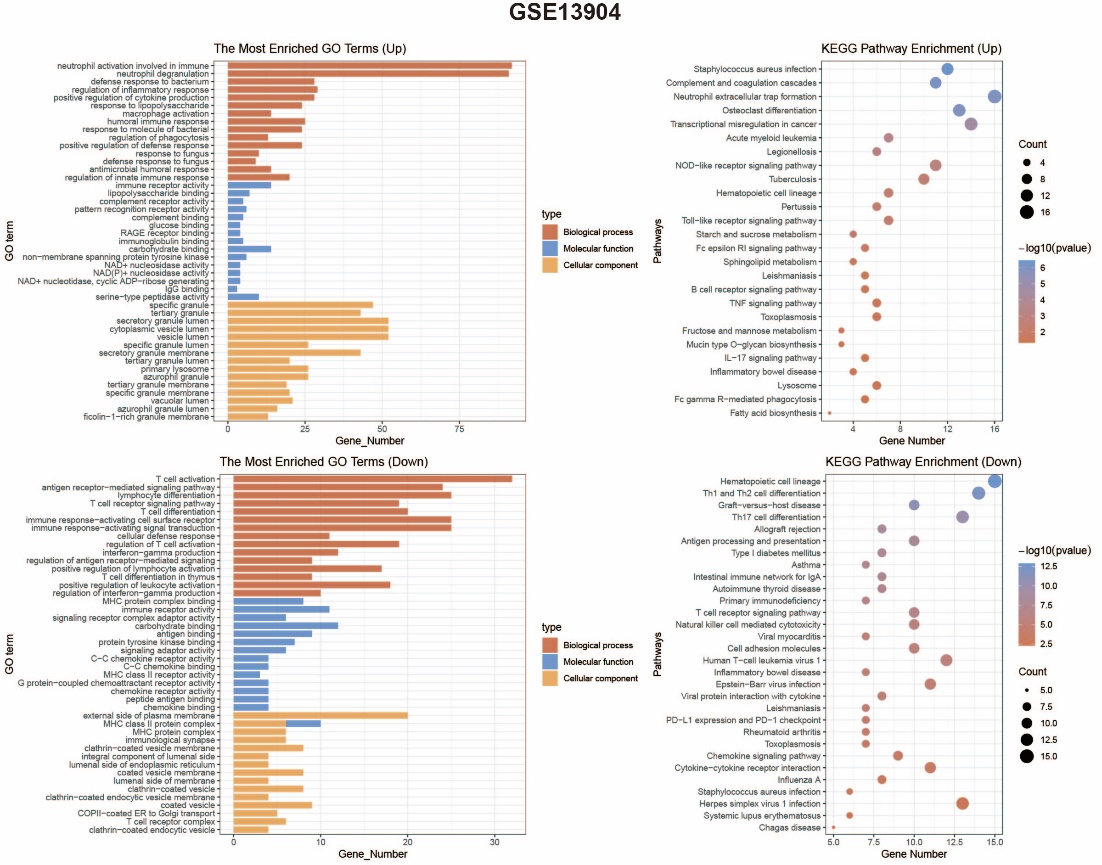


**Supplementary Figure 3** Functional enrichment analysis included KEGG pathway enrichment and GO term enrichment results of differential genes (**GSE13904**).

**Supplementary Figure 4**

**
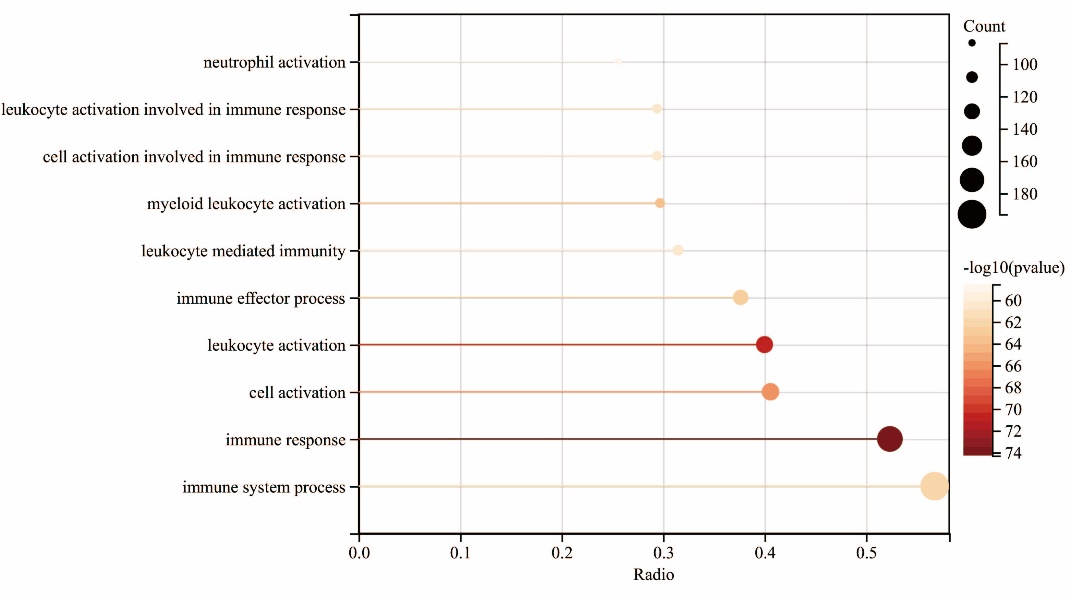
**

**Supplementary Figure 4** GO term enrichment results of differential genes. Venn analysis showed that 3 intersected genes (**GSE26378, GSE26440 and GSE13904).**

**Supplementary Figure 5**

**
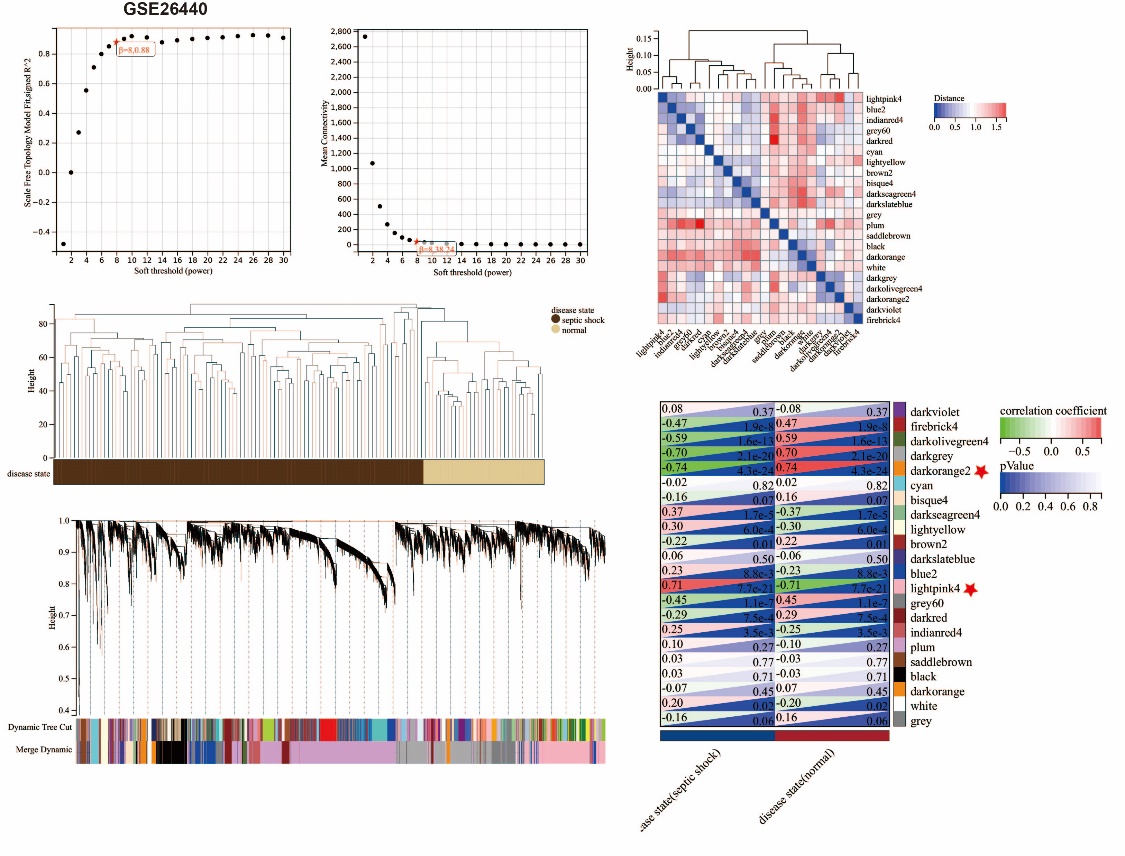
**

**Supplementary Figure 5** Construction of co-expression network for sepsis and health status in children in **GSE26440** dataset**.**

**Supplementary Figure 6**

**
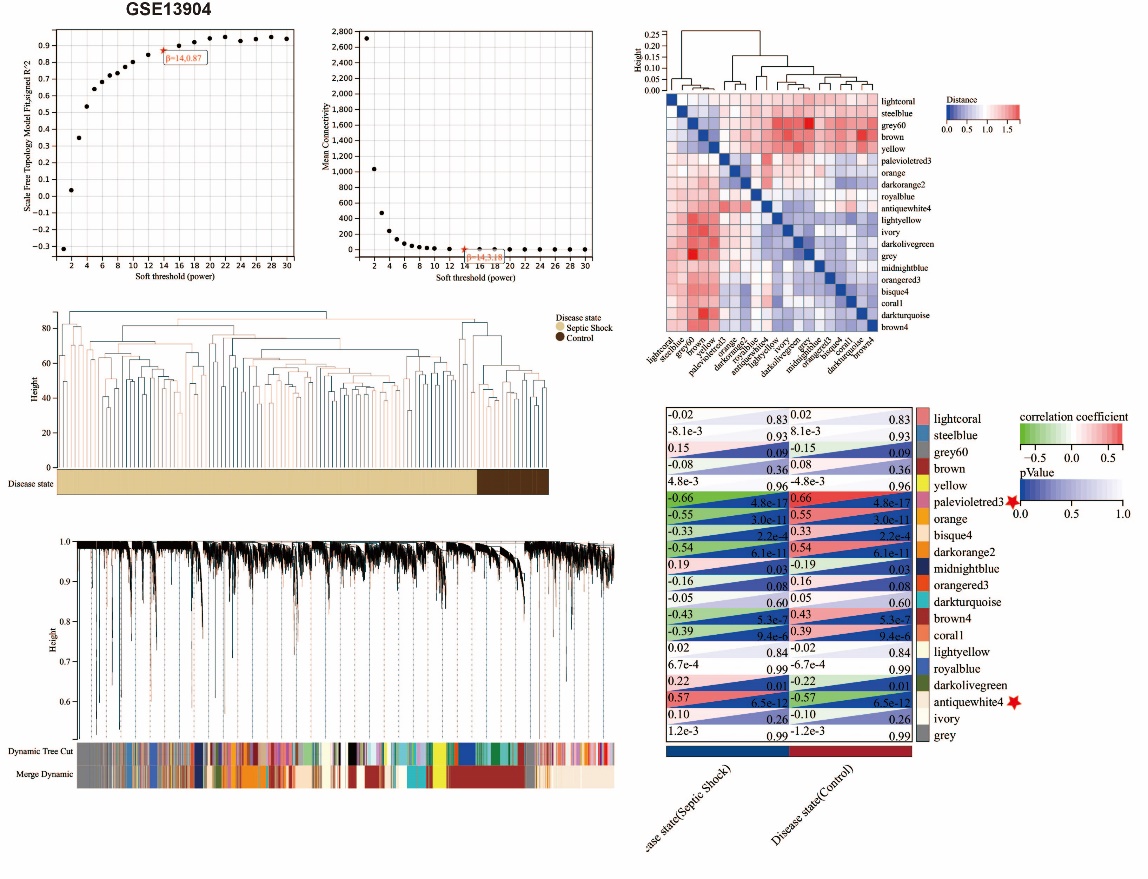
**

**Supplementary Figure 6** Construction of co-expression network for sepsis and health status in children in **GSE13904** dataset**.**

**Supplementary Figure 7**

**
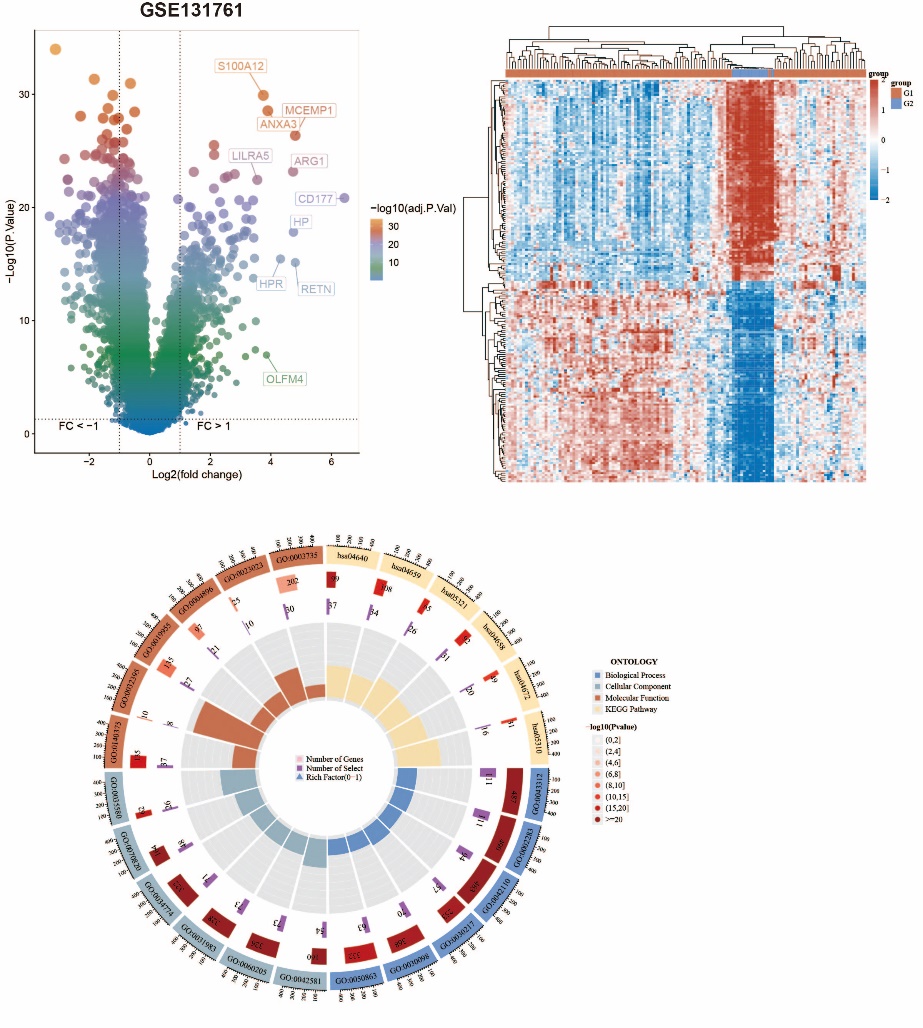
**

**Supplementary Figure 7** The differential genes and functional analysis in **GSE131761.**


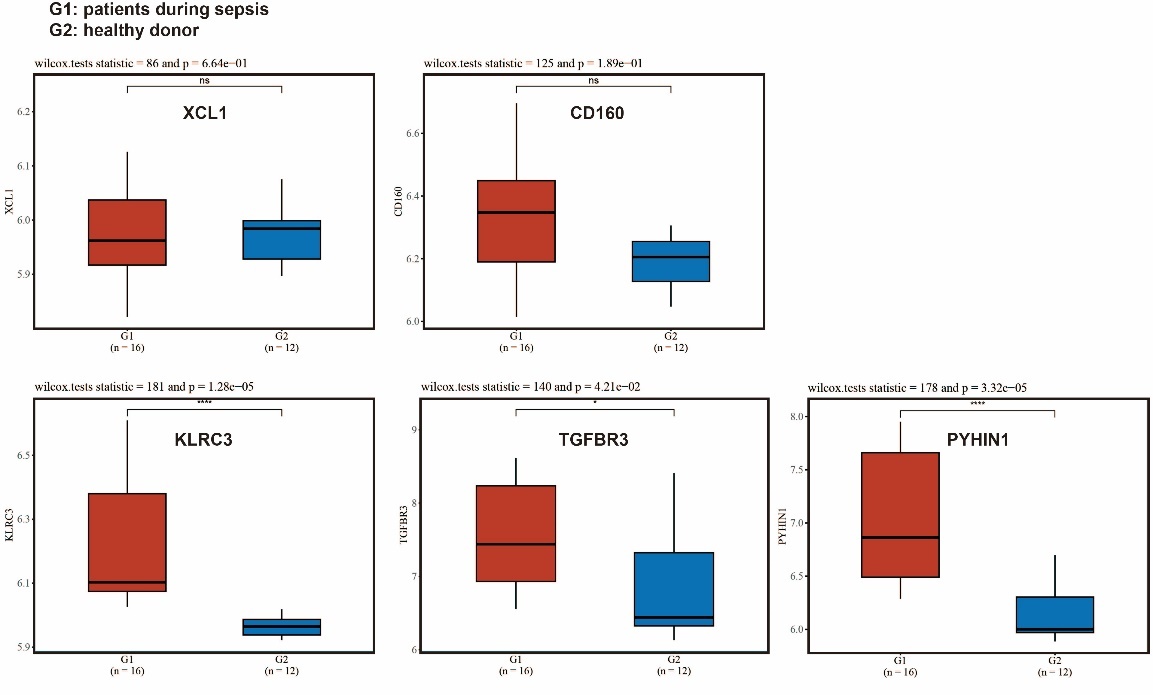


**Supplementary Figure 8** Differential expression analysis of the five genes (XCL1, CD160, KLRC3, TGFBR3 and PYHIN1) in **GSE46955.**

**Supplementary Method**

***Identification of the differentially expressed genes***

Gene expression profile files were generated by normalizing the data and correcting the expression value in batches using the "sva" package. The LIMMA package was utilized to identify DEGs between the pediatric sepsis group and the control group, and a volcano plot was produced to highlight the differential expression of DEGs. Adjusted P values were looked at in GEO to account for the possibility of false-positive outcomes. An adjusted P value < 0.05 and |log2FC|> 0.5 were considered to be the cutoffs for DEGs. Using R software's pheatmap package, a heatmap was generated based on the DEGs that had been screened.

***Functional enrichment analysis***

In order to conduct additional functional enrichment analysis, we are currently extracting genes from modules of interest. An examination using Gene Ontology, also known as GO, was carried out so that distinguishing features of the organism’s biological composition could be uncovered. In order to achieve a more in-depth comprehension of the functional characteristics, an investigation into the KEGG pathway enrichment database was carried out.

***GeneMANIA analysis***

GeneMANIA imports interaction networks from public databases and predicts these related genes for a query gene set. It integrates with Cytoscape for network visualization, analysis and automation. GeneMANIA will find these genes that may share functions with it based on their interactions with it, and analyze and predict the functions of those genes.

**Supplementary Table 1**

Clinical Information on microarray datasets obtained from GEO

| **GEO dataset** | Platform | **Sepsis (number)** | Normal (number) | **Age (Years)** |
| --- | --- | --- | --- | --- |
| **GSE26378** | GPL570 | **82** | 21 | **≤ 10** |
| **GSE26440** | GPL570 | **98** | 32 | **≤ 10** |
| **GSE13904** | GPL570 | **106** | 18 | **≤ 10** |
| **GSE131761** | GPL13497 | **115** | 14 | **27-92** |

*GEO* Gene Expression Omnibus
